# Farm characteristics affecting antibiotic consumption in pig farms in England

**DOI:** 10.1186/s40813-022-00248-z

**Published:** 2022-01-28

**Authors:** S. M. Matheson, S. A. Edwards, I. Kyriazakis

**Affiliations:** 1 School of Natural and Environmental Sciences, Newcastle University, Newcastle upon Tyne, NE1 7RU UK; 2 Institute for Global Food Security, School of Biological Sciences, Queen’s University, Belfast, BT7 1NN UK; 3 Present Address: Sanday Bioscience Ltd, Academy House, Shedden Park Road, Kelso, Roxburghshire TD5 7AL Scotland

**Keywords:** Animal resilience, Antibiotic use, Critically important antibiotics, Enrichments, Farm characteristics, Pig, Secondary databases, Straw-based systems, Ventilation

## Abstract

**Background:**

Pig production has been highlighted as one of the highest users of antibiotics amongst livestock, with several studies suggesting a variety of approaches to antibiotic reduction. We aimed to investigate links between antibiotic use (defined as total amount of critically (CIA) and non-critically important antibiotics, and as mg per kg of pig on farm), production stages present on farm (Breeder–Finisher, Nursery–Finisher, and Finisher), and pig farm characteristics using farm data collected through national recording systems in Great Britain for 2017 & 2018. Providing enrichment within pig pens may reduce the need for antibiotics by enhancing both pig welfare and resilience to infection; this was one of the hypotheses addressed by this paper.

**Results:**

The amount of antibiotic used, expressed as mg/kg, reduced between 2017 and 2018 for Breeder–Finisher farms, but not for Nursery–Finisher or Finisher farms. Breeder–Finisher farms were more likely to use CIA compared with other production stages. Larger farms were more likely to use CIA, but farm size had no effect on mg/kg of antibiotic used. As the proportion of pens containing straw increased, the total use of antibiotics decreased for Breeder–Finisher, but not for Nursery–Finisher or Finisher farms. As the proportion of pens containing straw increased, the probability of using CIAs also decreased. Farms with a higher proportion of finisher pens with an outdoor space had a lower use of non-critical antibiotics and lower probability of use of CIA. Farms with a higher proportion of pens with automatically controlled natural ventilation (ACNV) had lower total use of antibiotics, although ACNV had no effect on the probability of using CIA.

**Conclusions:**

We quantified the influence of farm characteristics on the consumption of antibiotics in pig farms in England. Our findings support the hypothesis that farm characteristics have an influence on antibiotic use within a system and suggest that this reflects the balance of effects on both animal resilience and disease challenge. Consistent with our hypothesis, provision of straw was associated with reduced antibiotic use. We also demonstrate the value of using secondary databases, although further structural improvements are required to facilitate effective database combination and ensure maximum information benefits can be realised.

## Introduction

Concerns over the use of antibiotic medication in farm livestock have intensified over the last decade. Antibiotic resistance is inherently associated with the overuse or misuse of antibiotics during agricultural practices [1], such as treatment of sick animals, growth promotion (although banned within the EU) [2], as well as disease prevention [3]. The main concerns regarding overuse or misuse by the livestock sectors are centred on the medication of animals with antibiotics that are also critically important for humans, such as third-generation cephalosporins and fluoroquinolones—antibiotics that are classified by the European Medicines Agency as category B-restricted [3]. Given the importance and interdependence of the human, animal, and environmental dimensions of antibiotic resistance, a One Health approach based upon linking human health and nutrition with animal and environmental health, seems a logical basis to reduce the problem by eliminating the inappropriate use of antibiotics [3]. A study tracking antibiotic resistance, in relation to antibiotic usage in five common pig pathogenic bacteria on Danish farms, found that antibiotic resistance for some pathogens varied over time in response to farm usage, thus emphasising the need for continuous surveillance of resistance patterns [4].

Within the EU-wide livestock sector, pig production has been highlighted as one of the highest-users of antibiotics [5, 6]. Intensive pig production can be more efficient than extensive production, and more investment can be made into bespoke housing, which can further improve animal health [7]. However, large animal populations can also lead to rapid distribution of pathogens and can potentially lead to large number of animals needing treatment [8, 9]. Antibiotics are relatively cheap to buy and therefore, historically, have been used prophylactically to prevent this happening on a wide scale. Recent understanding of antibiotic resistance has led to changes in practice and antibiotic use in the UK has reduced by 60% since 2015 [10].

Several methods have previously been suggested to help reduce the use of antibiotic treatments in pigs, such as improving farm management and increasing site biosecurity [11, 12], early detection and treatment of disease [13], and alternative treatments such as food additives (e.g., acidifiers [14]). Other studies have suggested that the provision of enrichment within the pen may also achieve a reduction in the need for antibiotics, through an enhancement of animal resilience. For instance, provision of environmental and social enrichment has been shown to improve welfare and reduce disease susceptibility in pigs [15]. Therefore, there is the possibility that antibiotic usage could be reduced by improving pig welfare via changes in the environment, with minimal loss of function and production. Enrichment investigations are usually performed at a small scale, due to their nature. Further investigation of this possibility at a farm level would require the monitoring of antimicrobial use over a period of time and preferably an investigation that involves many farms [11].

In the United Kingdom, farms are able to record their antibiotic use on the Agriculture and Horticulture Development Board’s (AHDB) electronic medicine book (eMB). Previously, in the UK, antibiotic sales were the only proxy available for estimating usage in the pig industry [15]. However, many products share a licence between pigs and other species, primarily chickens, therefore only using total sales resulted in an inflated and inaccurate picture of pig sector only use of antibiotic treatments. A farm usage reporting scheme should allow better monitoring of antibiotic use and allow producers, vets, and other stakeholders, such as researchers, to identify characteristics of usage and track how usage changes over time. If the antibiotic use data are paired with data on farm characteristics, and the incidence of health and welfare outcomes of the animals within the system, then one could explore questions about the farm characteristics that contribute to antibiotic use. Thus, the objectives of this paper were threefold: 1) to link data on antibiotic use in UK pig farms with other data on farm characteristics, 2) to investigate antibiotic use and identify the use by the different animal classes (production stages) on farm over a period of 2 years, and 3) to identify the farm characteristics that may be associated with a lower level of antibiotic use in pig farms.

## Materials and methods

### Data and data management

The data used in the analyses were collected for 48 months between January 2017 and December 2018 by means of the electronic medicine book for pigs (eMB-Pigs). Pig producers self-report their own antibiotic usage and upload the data to the eMB-Pigs database using the dedicated eMB-Pigs website (https://ahdb.org.uk/electronic-medicine-book-for-pigs-emb-pigs). Farm units submit antibiotic usage to eMB, on a quarterly basis, as a requirement of Quality Assurance schemes (see below). Although eMB-Pigs covers the entirety of the United Kingdom, the data available for use in these analyses were recorded on farms in England only, i.e., approximately 85% of UK pig finishing farms in 2018. Antibiotic usage was recorded as the usage, in kg, of active ingredient of each antibiotic (as defined by the Veterinary Medicines Directorate) for each farm for a specified return length.

Additional explanatory farm descriptors, collected in two separate scheme databases, were also used: the Real Welfare database (collated by AHDB) and the Red Tractor database (database collated by the Red Tractor Farm Assurance Scheme). The Real Welfare scheme (https://ahdb.org.uk/real-welfare) involves on farm assessment of pig welfare by veterinary surgeons using a set of five objective and repeatable measures known as welfare outcomes. Sample pens of finisher pigs (50 kg plus) are assessed for tail damage, body lesions, lameness and the provision presence of pigs in production pens which are in need of hospitalisation. An additional optional measure records the use of environmental enrichment by the pigs. On-farm assessment of welfare is carried out by protocol-trained large-animal veterinarians 2–4 times per year on finishing farms (with pigs > 50 kg) and, in addition to welfare outcome assessments, records the type of environmental enrichment available and other farm descriptors such as ventilation type and feeding practises in the finishing pen assessed. Farms participating in the Red Tractor Farm Assurance scheme (https://redtractor.org.uk) are certified against standards centred on traceability, food safety, animal health and welfare and environmental protection, and assessors collect some farm descriptor information during audits, including straw use within pens at all production stages.

Two studies were conducted, the first aimed to elucidate the relationship between antibiotic usage and farm characteristics in finishing herds (Real Welfare; RW) and the second to investigate the relationship between antibiotic usage and farm characteristics in breeding herds (Red Tractor; RT). Table 1 lists the relevant farm descriptors available for this study from the RW database and the RT database). Data were linked at farm level and each farm was given an anonymous identifier (unique ID) to ensure anonymity was maintained throughout the study.

**Table 1:** List of the farm descriptors, additional to the electronic Medicines Book, available in the Real Welfare (RW) and Red Tractor (RT) Farm Assurance schemes (bold descriptors were used in the final statistical models; non-bolded descriptors did not show significance in preliminary models and were not further utilised; please refer to the statistical analysis section for details)

Descriptor (assurance scheme)	Additional farm descriptors
Ventilation (RW)	Powered Natural Natural + fan **ACNV** (automatically controlled natural ventilation)
Building type (RW)	Indoor kennel + outside yard Indoor kennel only indoor open-plan with internal divisions between pens Indoor open-plan **Outdoor shelter and field** ‘Trowbridge style’ Other pen type
Feed availability (RW)	Ad libitum Restricted
Feed type (RW)	Meal Liquid Pellets
Feed delivery (RW)	Floor Hopper Trough
Pen enrichments (RW)	**Straw** Deep straw Shavings Cardboard Other
Pen bedding (RT)	**Grower pens with straw** Finisher pens with straw Dry sow pens with straw

### Data manipulation (pre-analysis)

The variables of interest for further analysis were developed from the records which investigated different aspects of antibiotic use. From the eMB data, we calculated the following variables:

*mg/kg (mg/kilogram of pig on farm)* The amount of mg of antibiotic used per kg of pig for each farm was calculated from the total amount of antibiotics used and standardised pig weights for each stage of growth present on the farm at the time of report submission, as shown in Eq. 1. To scale each farm up to a yearly submission, the total usage and the numbers for each submission period (the number of slaughter pigs leaving farm, breeding pigs leaving farm, weaners/growers leaving farm, and the number of weaned piglets leaving farm) were all summed to get a yearly figure. The average number of sows/boars present on farm were then calculated for each farm for the year. The yearly mg/kg per farm was then calculated using the formula contained in Eq. 1.

$${\text{mg/kg}} = \frac{{{\text{Total}}\,{\text{usage}} \times 1,000,000}}{\begin{aligned} &{\text{annual}}\,{\text{average}}\,{\text{number}}\,{\text{of}}\,{\text{sows/bars}}\,{\text{on}}\,{\text{farm}}\, \times 240 + ({\text{breeding}} + {\text{slaughter}}\,{\text{pigs}}\,{\text{leaving}}\,{\text{farm}}) \times 65 \\ &\quad + ({\text{weaners/growers}}\,{\text{leaving}}\,{\text{farm}}) \times 25 \\ &\quad + ({\text{piglets}}\,{\text{leaving}}\,{\text{farm}}) \times 4 \\ \end{aligned} }$$

The calculation used to estimate the mg of antibiotic used per kg of pig on farm. For the period that the pigs are on farm, the average weight of breeding sows and slaughter pigs is estimated to be 65 kg; the average weight of weaner/growers is estimated to be 25 kg; the average weight of piglets is estimated to be 4 kg [16].

*Total amount of non-critically important antibiotics used (kg)* The total amount of non-critically important antibiotics (non-CIAs) was calculated by summing all the non-CIA data. This variable allows a crude overall measurement of the amount of antibiotics used.

*Total amount of critically important antibiotics (kg)* the total amount of critically important antibiotics (CIAs) was calculated by summing all the amount of polymixins (colistin), cephalosporins (3rd & 4th generations) and fluoroquinolones [6]. The usage of CIAs was sparse, so these data were refined at farm level to a binary state (yes—used CIAs, no—did not use CIAs). This variable specifically concentrates on the antibiotics most important for human medicine, and as such, which need to be reserved for human use.

For the farm descriptors, as the farm systems in this dataset were often not homogeneous, the pen level data provided by Real Welfare were converted into the proportion of pens with a specific characteristic on each unique farm. For instance, for Real Welfare, the proportion of pens with automatically controlled natural ventilation (ACNV) was calculated. For Red Tractor, the farm level data provided allowed farms to be classified as having pens with bedding as a yes/no binary variable for each production stage. This action was performed for all of the descriptors available (Table 1).

For the data descriptors of farm category, return length (i.e., number of months), and year, there was a problem that the return length was not the same for all farms and farm category was not consistent throughout the year. It should be noted that farm category was self-reported information and may not accurately represent the category of the farm encompassing the whole year, e.g., some farms recorded four submissions per year, but labelled each submission in different categories (i.e., Breeder, Nursery, Gilt Unit, and Finisher). For submission return-length, most farms submitted data in 3-month return length; however, other farms returned 6-month or 12-month returns with this multiple labelling of categories resulting in there being fewer total farms in the raw dataset than the sum of the farms in all categories. Thus, the self-reported farm category may differ within farm depending on the time of year, causing problems when upscaling to a yearly record.

To eliminate inconsistencies caused by farms having multiple category-labels throughout the year, new farm categories were assigned depending on the reported presence of different pig growth stages on farm during the entire year (i.e., upscaling the category label to a yearly label; Table 2). Breeder farms recorded breeding sows/boars and piglets, but no slaughter pigs and no grower or weaner pigs. Breeder-Weaner farms recorded sows/boars, piglets and weaner/grower pigs, but no slaughter pigs. Breeder–Finisher farms recorded all stages. Nursery farms recorded weaners/growers, but no sows/boars or finisher stages. Nursery–Finisher farms recorded weaners/growers and Finisher pigs, but no sows/boars and no piglets. Finisher units recorded only finisher pigs. Please note that Gilt Units are indistinguishable from Finisher units when categorising by using only presence/absence of specified growth stages as the input data.

**Table 2:** The total number of records in each production category in the raw eMB-Pigs database for 2017 and 2018 after dataset linking with the Real Welfare database. Data were up-scaled to a yearly basis and the farm categories re-labelled

Farm category	No. records 2017	No. records 2018	Total records
Breeder only	14	7	21
Breeder–Finisher	294	258	552
Breeder–Weaner	7	4	11
Finisher	817	768	1585
Nursery	4	7	11
Nursery–Finisher	80	107	187

### Database linking

*Study 1: the relationship between antibiotic usage and farm characteristics for ****finishing herds****.* There were 1800 unique farm IDs with 11,004 records in the eMB database before database combining. There were 1459 unique farm IDs with 58,668 pen records in the Real Welfare finishing herd database before database combining. When eMB and Real Welfare were upscaled to a year and combined, there were 1370 matching unique farm IDs with 2323 records. Data analysis was only performed on those categories of farm recording the finishing stage (Breeder–Finisher, Nursery–Finisher, and Finisher) due to low numbers of records for the other categories.

*Study 2: the relationship between antibiotic usage and farm characteristics for ****breeding herds***. There were 1814 unique farm IDs with 2232 records in the Red Tractor breeding herd database before database linking. However, when eMB and Red Tractor were combined there were only 98 matching unique farm IDs with 114 records. Data analysis was only performed on those categories of farm recording the breeding stage (Breeder, Breeder–Finisher, and Breeder-Weaner), to complement the Real Welfare analysis.

### Statistical analysis

Preliminary analyses were performed including all farm descriptors, with the non-significant factors removed in a process of model reduction; all interactions were investigated and then removed if found not to be significant. Therefore, for the final model in each study, aside from the base model of farm size, farm category, year, and the interaction between farm category and year, only additional farm descriptors of significance were included.

#### Study 1. eMB and Real Welfare databases—finisher stage focused

All analyses of the mg/kg and the total amount non-critically important antibiotics used, the generation of predicted means for the fixed effects, and covariance parameters for covariates, were performed using the Glimmix procedure (generalised linear mixed model) in SAS 9.4, using the Tukey–Kramer adjustment for multiple comparisons. The fixed effects used in the model were farm category, year, and the interaction between category and year. The covariates used, following model reduction after preliminary analyses, were the sum of the number of finisher pigs (as a proxy for farm size), the proportion of pens with an outdoor space (propOUT), the proportion of pens with automatically controlled natural ventilation (propACNV), the proportion of pens with straw available (propSTRAW), and the interaction between farm category and propSTRAW. The random effects were the residual blocked with the farm ID.

The analysis of the probability of using CIAs, the generation of predicted means for the fixed effects, and covariance parameters for covariates, were performed using the Glimmix procedure (generalised linear mixed model) with the binary response distribution and Logit link function in SAS 9.4, using the Tukey–Kramer adjustment for multiple comparisons. The fixed effects used in the model were farm category, year, and the interaction between category and year. The covariates used were the sum of the number of finisher pigs (as a proxy for farm size), the proportion of pens with an outdoor space (propOUT), the proportion of pens with automatically controlled natural ventilation (propACNV), the proportion of pens with straw available (propSTRAW), and the interaction between farm category and propSTRAW. The random effects were the residual blocked with the farm ID.

#### Study 2. eMB and Red Tractor databases—breeder stage focused

As the proportions of pens with straw within farm was not available for the Red Tractor dataset, all analysis was performed on whether straw bedding was available, or not for a given pig category at the farm level. All analysis of the mg/kg, the generation predicted means for the fixed effects, and covariance parameters for covariates, were performed using the Glimmix procedure (generalised linear mixed model) in SAS 9.4, using the Tukey–Kramer adjustment for multiple comparisons. Following model reduction after preliminary analyses, the fixed effects used in the model were year and the grower stage with straw pen bedding or not (bedYN). The random effects were the residual blocked with the farm ID.

## Results

### Study 1. eMB and Real Welfare databases—finisher stage focused

Nursery–Finisher farms had a higher mg/kg of antibiotics used in comparison to other farm categories, although there was no significant difference between Breeder–Finisher and Finisher farms in mg/kg used (mean ± sem, Nursery–Finisher—118.33 ± 10.8004, Breeder–Finisher—88.60 ± 7.6647, Finisher—84.57 ± 3.7545, *P* < 0.001). There was no significant difference between the years in the use of antibiotic mg/kg (2017: 100.59 ± 5.7370, 2018: 93.75 ± 5.3620, *P* > 0.05).

**Fig. 1:**
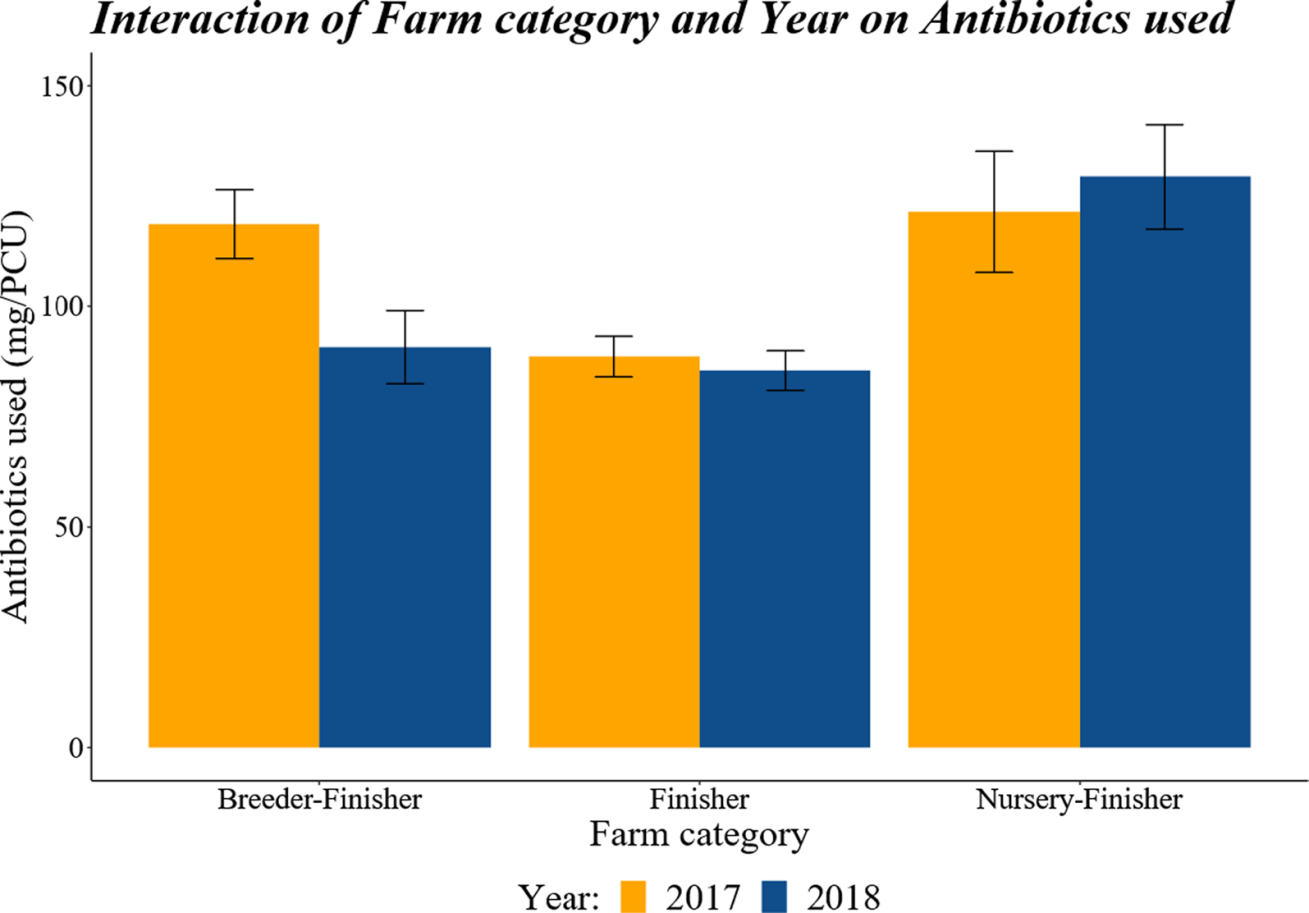
The effect of farm category and year on the mg/kg of antibiotics used between the years 2017–2018. There were no differences between years for either the Nursery–Finisher or Finisher farm categories, but there was a significant difference between years for the Breeder–Finisher farms

There was a significant category x year interaction in the mg/kg of antibiotics used (*P* = 0.0092; Fig. 1) due to a significantly higher mg/kg within used the Breeder–Finisher farms in year 2017 compared with 2018 (*P* = 0.007); there were no significant differences between years in mg/kg used for either the Nursery–Finisher or Finisher farm categories.

**Fig. 2:**
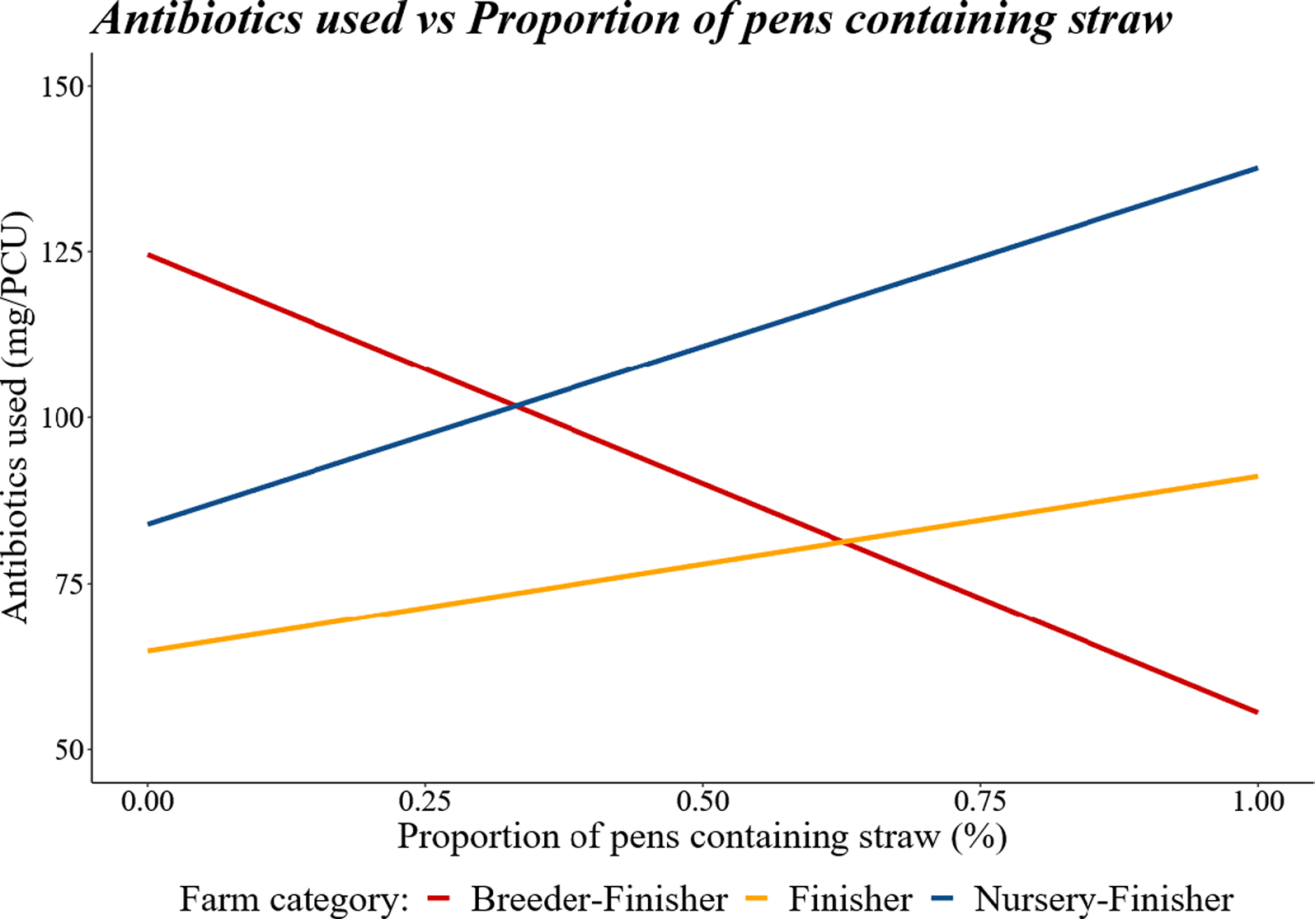
The effect of farm category on the regression relationship between the proportion of pens containing straw and the mg/kg of antibiotics used during the years 2017–2018

Farm size had no effect on the mg/kg of antibiotics used (regression coefficient ± sem, 0.000697 ± 0.000697, *P* > 0.05). Farms with a higher proportion of finishing pens with an outdoor space had a lower mg/kg of antibiotics used (regression coefficient ± sem, − 68.3354 ± 17.7186, *P* = 0.0001). Farms with a higher proportion of ACNV pens had a lower mg/kg of antibiotics used (regression coefficient ± sem, − 26.2319 ± 9.3383, *P* = 0.0051). The proportion of pens containing straw had no effect on the mg/kg of antibiotics used on its own (*P* > 0.05); however, there was an interaction between propSTRAW and production category (Fig. 2, *P* < 0.001) with mg/kg of antibiotics used increasing as propSTRAW increased for Nursery–Finisher and Finisher category farms but decreasing as propSTRAW increased for Breeder–Finisher farms.

Breeder–Finisher farms had a higher probability of using CIAs when compared with the other farm categories, while there was no significant difference between Nursery–Finisher and Finisher farms (mean ± sem, Breeder–Finisher: 0.336 ± 0.0272, Nursery–Finisher: 0.108 ± 0.0246, Finisher: 0.064 ± 0.0072, *P* < 0.001). There was no significant difference in the probability of CIA use between 2017 and 2018 (2017: 0.156 ± 0.0187, 2018: 0.123 ± 0.0135, *P* > 0.05) and there was no significant interaction between farm category and year on the probability of using CIAs.

As the farm size increased, the probability of using CIAs increased (regression coefficient ± sem, 0.000076 ± 0.000013, *P* < 0.0001). Farms with a higher proportion of finisher pens with an outdoor space had a lower probability of using CIAs (regression coefficient ± sem, − 1.6226 ± 0.5851, *P* = 0.0057), whereas the proportion pens with ACNV had no effect on the probability of CIAs (regression coefficient ± sem, − 0.00574 ± 0.2666, *P* > 0.05). As the proportion of finisher pens containing straw increased, the probability of using CIAs decreased (regression coefficient ± sem, − 0.6534 ± 0.5741, *P* = 0.0378), however, there was no interaction between category and the proportion of pens with straw in the probability of using CIAs.

Finisher farms had lower total usage (kg/year) of antibiotics when compared with the other categories, while there was no significant difference between Breeder–Finisher and Nursery–Finisher (mean ± sem, Finisher: 27.65 ± 2.3466, Breeder–Finisher: 59.18 ± 4.4149, Nursery–Finisher: 52.99 ± 6.5014, *P* < 0.001). Year differences were significant, as 2017 had a significantly higher usage of antibiotics (kg/year) when compared with 2018 (2017: 50.86 ± 3.8785, 2018: 42.35 ± 2.7705, *P* > 0.05).

**Fig. 3:**
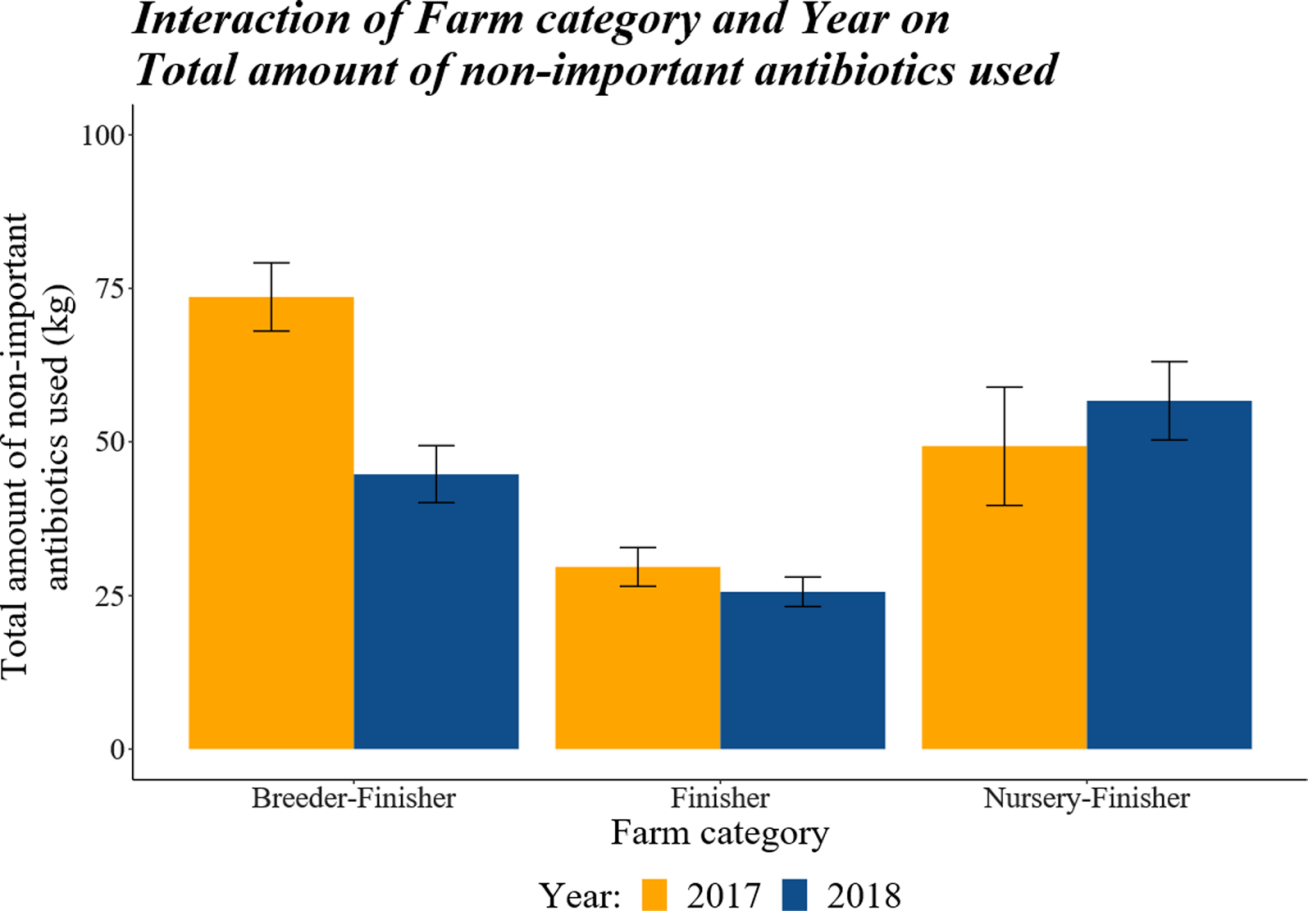
The effect of farm category and year on the total amount of non-critical antibiotics used (kg) between the years 2017 and 2018. There were no differences in the total amount of non-critical antibiotics used between years for either the Nursery–Finisher or Finisher farm categories, but there was a significant difference between years for the breeder–Finisher farms

There was a significant interaction between farm category and year in the total usage (kg/year) of antibiotics (*P* < 0.0001; Fig. 3), which was accounted for by Finisher farms using significantly less antibiotics in comparison to Breeder–Finisher farms in both 2017 (*P* < 0.0001) and 2018 (*P* = 0.0031) and with Nursery–Finisher farms in 2018 (*P* < 0.0001). Breeder–Finisher farms also used less non-important antibiotics in 2018 compared with 2017 (*P* < 0.0001).

**Fig. 4:**
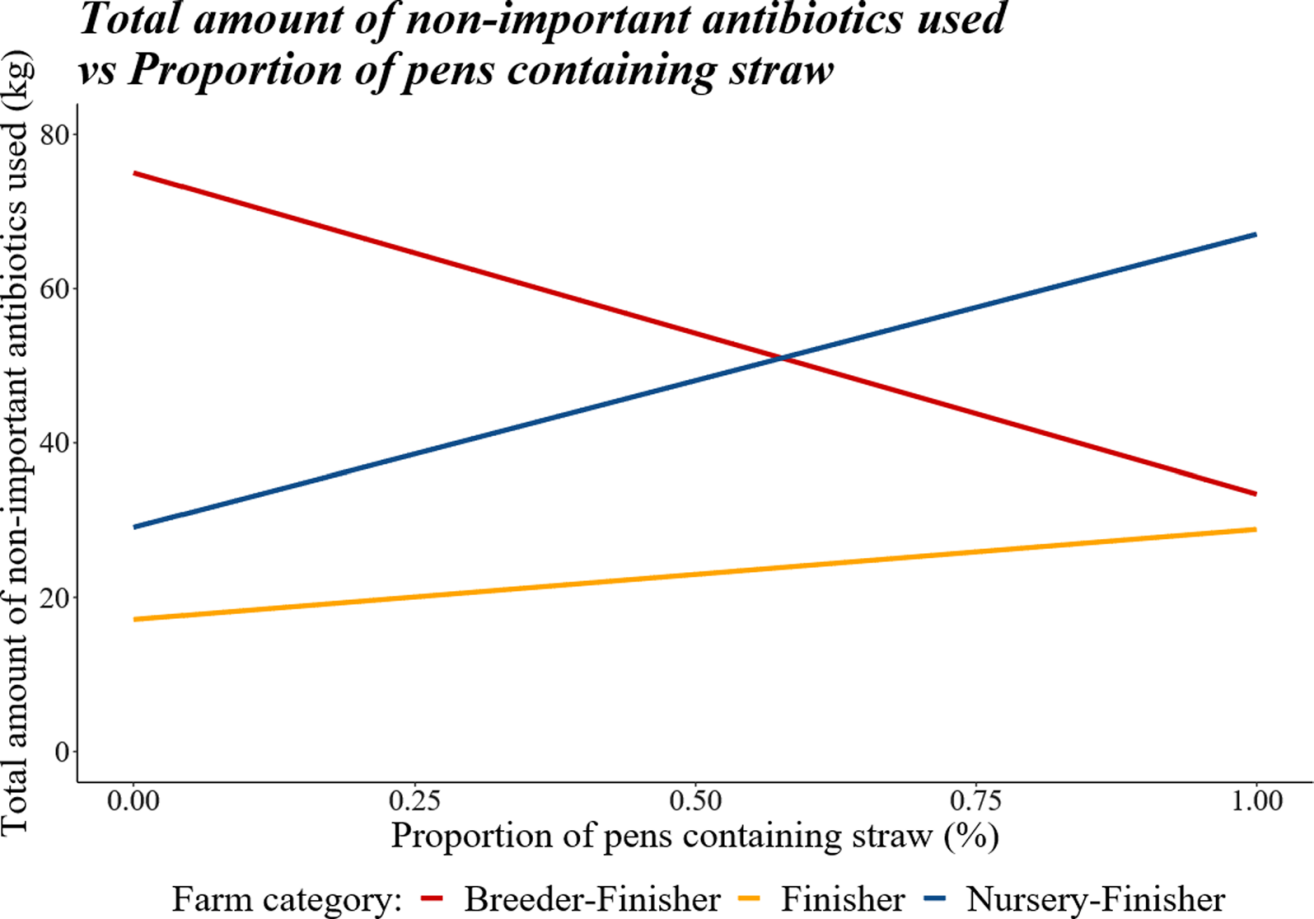
The effect of farm category on the relationship between the proportion of pens containing straw and the total amount of non-critically important antibiotics used between the years 2017–2018

As the farm size increased, the usage of non-critical antibiotics increased (regression coefficient ± sem, 0.006666 ± 0.000387, *P* < 0.0001). After accounting for farm size, farms with a higher proportion of finishing pens with an outdoor space had lower usage of non-critical antibiotics (regression coefficient ± sem, − 28.4176 ± 10.1221, *P* = 0.0051). Farms with a higher proportion of ACNV pens had lower usage of non-critical antibiotics (regression coefficient ± sem, − 18.1636 ± 7.8088, *P* = 0.0202). The proportion of finishing pens containing straw had no effect on its own; however, there was an interaction between propSTRAW and category (Fig. 4, *P* < 0.001) with total usage of non-critical antibiotics increasing as propSTRAW increased for Nursery–Finisher and Finisher categories but decreasing as propSTRAW increased for Breeder–Finisher farms.

### ***Study 2***—***eMB and Red Tractor databases***—***breeder stage focused***

For the smaller number of farms in this study, there was no significant difference between the years in the mg/kg of antibiotics used (2017: 112.13 ± 17.0911, 2018: 104.54 ± 28.1517, *P* > 0.05). Farms which had grower pens with straw bedding had a higher mg/kg of antibiotics used compared with grower pens without straw (Yes: 139.46 ± 21.7737, No: 77.21 ± 24.8983, *P* = 0.0477). Categorisation on use of straw in other production stages showed no significant effect on the mg/kg of antibiotics used outcome.

The models for the variables Important antibiotics used (yes/no) and Non-critical antibiotics (total kg used) did not converge, and are therefore not reported.

## Discussion

Here we describe the relationship between the characteristics of pig production systems and antibiotic use in the English pig sector over a period of 2 years. We hypothesised that antibiotic usage could be reduced by systems which enhance the resilience of the animals by improving their ability to cope with environmental challenges, which includes pathogen challenge, with a minimal loss of function and production [17]. However, we recognise that production system characteristics may also alter the level of pathogen challenge presented to the animals [18]. Thus, this study aimed to look at the relationship between antibiotic usage in the pig sector and the farm characteristics within the system.

Public concern and political pressure for a reduction in the consumption and prescription of antibiotics in agriculture has been increasing [19]. This concern led the UK pig production sector to address a reduction in antibiotic use and, since 2015, the amount of antibiotics used by the pig industry has reduced by over 60% (to 2019), while the amount of critically-important antibiotic use has fallen by 75% [15]. The UK is currently the fifth-lowest user of antibiotics for food-producing animals (using mg of antibiotic sold corrected for livestock population) among more commercially productive European countries [20], with good progress made, as highlighted in the 2017–2020 RUMA’s Targets Task Force report [10]. Pig specific targets were to i) reduce overall use to 99 mg/kg by 2020, and ii) for the highest priority antibiotic use to stay below specified levels. Target i) is close to being achieved, with reports of 104 mg/kg for quarter 1 & 2 in 2020, whereas target ii) was achieved in 2019 [10]. The data shown for the sample of farms in this study are similar to the targets reported.

Given the number high uptake of use of the databases, data from relatively few farms was able to be linked. This was largely due to missing data in the secondary databases. Secondary data is a data set collected for a different purpose, and, by default, the data it provides does not exactly match the current study design. Combining data may lead to missing data values and thus has the potential to impair data quality and the ability to perform the necessary analysis [21]. Despite these shortcomings, the current study is a relevant exercise of combining datasets with disparate characteristics and demonstrating how advantageous linking these datasets can be.

One issue that arose during this study was that the Real Welfare database should have no information regarding breeder or nursery farms, since inspections are only performed on the finishing stage. That some farms were classified as being breeder or nursery may have been due to the re-classification step during the preparatory data-cleaning stages. Since farms self-report their category into the e-Medicines website for each return-length, it is probable that some multi-stage farms (e.g., breeder–Finisher or nursery–Finisher) only entered antibiotic-use data when they were at a breeding stage of the cycle and then did not update the e-Medicines Book after that point. Thus, during re-classification to correct this multi-category information, the only information available was as breeder or nursery. This challenge has previously been identified in other studies when connecting data from multiple different sources [21, 22]. Additionally, no pen-level information was available regarding the number of pigs housed in each of the pen types recorded. This information would have allowed the correlation of the mg/kg of antibiotics to be related to the proportion of pigs housed in pens with straw, which would have provided greater accuracy than the data on number of pens of each type present on farm.

A further issue to be noted is that the results presented here for the mg of antibiotics used per kg of pig and year differs slightly from the AHDB published data for England overall [15]. There are several potential reasons for this difference:

1. The dataset used in this study was a subset of the total dataset available to AHDB. We only included farms where there were complete data sets in both eMB and Real Welfare databases.
2. As noted in the pre-analysis manipulation, to eliminate inconsistencies caused by farms having multiple category-labels throughout the year, new farm categories were assigned depending on the reported presence of different pig production stages on farm during the entire year (i.e., upscaling the category label to a yearly label). This will have resulted in slightly different numbers of farms in each category in each year. This study was performed on yearly data, whereas the AHDB published data performs analysis on the quarterly data where the farm category inconsistency is not apparent.
3. The explanatory variables of interest in this analysis will differ from the variables of interest on the full, quarterly, dataset. Linking the eMB and Real Welfare datasets allowed investigation of a different set of variables not available to the eMB-only dataset. Therefore, the multivariate models used allowed possible confounds like the proportion of pens with straw or the proportion of pens with ACNV to be incorporated in the assessment of an effect.

As has been noted, there were only 2 years of data available at the time of this study. As time progresses, this dataset will increase and provide more information about the scale and direction of antibiotic use, allowing for more in-depth, and interconnected, longitudinal studies. Secondary data from different sources would also allow further investigation of non-finishing stage farms, which are not included in the Real Welfare inspection programme, linking biosecurity, health, welfare and performance [22]. Self-reported classification was shown to be important information. More detail about the overall yearly cycle of each farm in addition to the stage captured in each submission would allow greater clarity on associations between stage of growth and antibiotic usage. Antibiotics are more commonly used during lactation/suckling and post-weaning periods [23], however, the e-MB does not capture the data relating to the stage at which antibiotics were administered amongst those present on the farm.

The farm characteristic that had a significant effect on antibiotic use in study 1 was provision of straw. As previously mentioned, enrichment investigations are usually performed at a small scale, due to their nature, and studies investigating the provision of different or multiple enrichments on a large-scale, farm-wide basis are currently lacking. It is therefore, unsurprising that the most common environmental enrichment available to include in this study was straw. Farms that had a higher proportion of pens containing straw were associated with a lower probability of using critically important antibiotics, although CIA use is now so low as to be negligible. There was no effect of the provision of straw alone for the total amount (kg) of non-important antibiotics used, nor the amount of antibiotics used per kg of pig present on farm (mg/kg), although there was an interaction between farm category and the proportion of pens containing straw for both variables. On Breeder–Finisher farms, weaned piglets are moved to pens on-site, where the microbial environment and enteric challenges are potentially familiar. In contrast, Nursery–Finisher farms have piglets arriving from (potentially multiple different herds) off-site, thus presenting weaned piglets with a novel physical and microbial environment and unfamiliar conspecifics in addition to the effects of transport, resulting in increased stress and enhanced challenges to the immune system [24]. The process of mixing conspecifics is noted as an inducer of stress and triggers behavioural, neuroendocrine, and immunological responses [25], whilst the mixing of animals from different sources is a major trigger for infectious issues. Unfortunately, the secondary dataset did not allow for such variables as whether pigs from different sources were mixed to be included in the model presented in this study. A granular secondary dataset would be required to tease apart the effects of piglet mixing and transportation on the amount of antibiotics used.

Pigs with access to straw enrichment are more active than pigs in slatted systems [26]. Environmental enrichment may satisfy their behavioural need to explore, resulting in lowered stress levels, and, thus, potentially influencing their immune response and status [27, 28]. Acute phase proteins are elevated in animals subjected to infection, inflammation or stress [29] and pigs from straw-bedded systems have been shown to have lower levels of acute phase proteins at slaughter [26]. Pigs born into less enriched housing also have lower humoral immune responses, although this effect may be overruled in later life if pigs are subsequently housed in an enriched environment [30]. The above, therefore, suggests that provision of environmental enrichment (including, but not limited to straw) for pigs not only improves their welfare, but also their resistance and resilience to infections, and improves the clinical outcomes for pigs as well as improving economic viability for farmers. Straw has often been considered the optimal solution for enrichment in pigs, however, there may be limitations, such as the requirement for solid flooring, for using it in large amounts [31] and other enrichments, such as wood, root vegetables, jute sacks, or hemp ropes [32, 33], should be potential alternatives for future research. Ultimately, the choice of the type of environmental enrichment would be dictated by the characteristics of the pig system to be used.

Straw, or other organic materials, may also have a beneficial effect by manipulating the development of the gut microbiota and their influence on immune competence, potentially reducing the need for antibiotics [34]. Microbiota composition can regulate the digestion and absorption of nutrients, and resistance to pathogen colonisation [35]. For example, straw has been found to reduce the proportion of Firmicute to Bacteroidetes found in the pig gut [36]. Beneficial species in gut microbiota prevent the multiplication of pathogens by simple competition for available nutrients [36] and also stimulate the immune system in piglets [37]. Other organic enrichment products may potentially elicit a similar response, although further experiments would be required for confirmation. A recent study using straw, moist peat, wood shavings, jute bags, and branches of a broom as environmental enrichment found that this enrichment positively drives important aspects of the development of the immune system and the establishment of gut microbiota in early life [34].

On-farm biosecurity may also contribute the reduction of antibiotic use [38]. Biosecurity may be considered as external, preventing the introduction of pathogens to the unit, or internal, the prevention of spread within the unit [38]. External biosecurity measures may include selective purchasing and quarantine of new animals [38], the establishment of clean (internal) and dirty (external) areas [39], and minimising risk of contaminated materials entering the clean area. Internal biosecurity measures may include testing water supplies for pathogens [40], and an all-in/all-out housing system in conjunction with thorough cleaning of pens with soap, water, and disinfectant [41]. These measures may be more difficult to apply in some types of straw-based systems. In contrast to Breeder–Finisher farms, antibiotic use increased in straw-based pens in herds that had growing pigs, but no breeding pigs on site. Weaned piglets moving to Nursery–Finisher /Finisher farms are placed in unfamiliar territory with potentially more mixing with non-familiar conspecifics and novel environmental and enteric challenges [24]. Under these conditions of enhanced challenge, being housed on solid flooring where contact with excreta is greater may result in increased risk of pathogen proliferation, requiring greater pharmaceutical intervention. Furthermore, high levels of dust, which can be generated by straw use [42], have been associated with increased levels of respiratory disease [43–45], while the temperature of the finishing house may combine with wet bedding to increase pathogen-load [46].

Farms that had a higher proportion of finishing pens with an outdoor area used a lower total amount of non-important antibiotics, had a lower rate of mg/kg usage of antibiotics, and had a lower probability of using critically important antibiotics. It should be noted that less than 4% of farms (87 out of 2343) had pens with access to an outdoor space, as this is not a common finishing system in conventional pig production in the UK, being mainly associated with niche marketing schemes. Pigs in such systems, as well as experiencing a more enriched environment which may change the gut flora, generally have greater floor and air space allowance, which may dilute the level of pathogen challenge [47].

In this study, pens with automatically controlled natural ventilation were associated with lower usage of antibiotics, both in absolute terms and when comparing the amount used per kg of pig. Automatically controlled natural ventilation is a type of mechanically-controlled system whereby the degree of opening and closing of ventilation apertures are determined by temperature sensors in the room [48]. Non-mechanical building ventilation has been associated with higher rates of respiratory disease, lower welfare scores, and lower rates of reproductive performance when compared to mechanically ventilated buildings [18, 49], which may also be attributable to a lower range of temperature and humidity fluctuations and control of toxic and noxious gases [50–53], and the accumulation of pathogens [18]. The survival of airborne respiratory pathogens depends on air humidity, while high levels of ammonia in the air enhances the attachment of respiratory pathogens [54].

Our results demonstrate pigs housed in buildings with automatically ventilated airflow which may reduce the build-up of pathogens and ammonia, and in pens with access to enrichment provided by straw, are associated with a reduction in antibiotic usage in the Breeder–Finisher farms. This concurs with a recent study where it was recognised that good management practices, low stocking densities, and a high health status were associated with low antimicrobial use [55]. The UK has unique animal husbandry and management systems, placing its pig sector in a unique position in comparison to other European countries [55]. However, the wide variety of management systems available in the UK, and represented in this study, does introduce confounding factors into the degree of pathogen challenge faced by the animals. It could be argued that these associations show either increased resilience, resistance, or a reduction in the pathogen load any individual animal was exposed to. Nevertheless, cost-effectiveness analysis assessing the economic impact of reduction of antimicrobial use coupled with improved biosecurity and increased vaccinations has shown that reducing antimicrobial use while implementing management change does not affect, and even increases profitability [56].

## Conclusions

Our findings support the hypothesis that farm characteristics have an effect on antibiotic use within a system. We hypothesise that antibiotic usage could be reduced by improving the ability of the animal to cope with environmental challenges, including infection challenge, with minimal loss of function and production. Confounding factors within each farm system may potentially affect the challenge faced by the animals, as demonstrated by the interaction between straw and system, which was positive for Breeder–Finisher s but negative for Nursery–Finisher and Finisher systems. Our results are derived from a multivariate model so that possible confounds like enrichment/ACNV are incorporated in the assessment of effect. Finally, our study also demonstrates the value of using, and linking, secondary databases although further structural improvements are required to facilitate effective database combination and ensure the maximum benefits can be realised.

## Data Availability

The data are owned by the respective owners (eMB-Pigs, Real Welfare, and Red Tractor).
